# Testing the reliability and ecological implications of ramping rates in the measurement of Critical Thermal maximum

**DOI:** 10.1371/journal.pone.0265361

**Published:** 2022-03-14

**Authors:** Chi-Man Leong, Toby P. N. Tsang, Benoit Guénard

**Affiliations:** School of Biological Sciences, The University of Hong Kong, Hong Kong SAR, China; Universidad de la Republica Uruguay, URUGUAY

## Abstract

Critical Thermal maximum (CT_max_) is often used to characterize the upper thermal limits of organisms and represents a key trait for evaluating the fitness of ectotherms. The lack of standardization in CT_max_ assays has, however, introduced methodological problems in its measurement, which can lead to questionable estimates of species’ upper thermal limits. Focusing on ants, which are model organisms for research on thermal ecology, we aim to obtain a reliable ramping rate that will yield the most rigorous measures of CT_max_ for the most species. After identifying three commonly used ramping rates (i.e., 0.2, 0.5 and 1.0°C min^-1^) in the literature, we experimentally determine their effects on the CT_max_ values of 27 species measured using dynamic assays. Next, we use static assays to evaluate the accuracy of these values in function of the time of exposure. Finally, we use field observations of species’ foraging activities across a wide range of ground temperatures to identify the most biologically relevant CT_max_ values and to develop a standardized method. Our results demonstrate that the use of a 1°C min^-1^ ramping rate in dynamic assays yields the most reliable CT_max_ values for comparing ant species’ upper thermal limits, which are further validated in static assays and field observations. We further illustrate how methodological biases in physiological trait measurements can affect subsequent analyses and conclusions on community comparisons between strata and habitats, and the detection of phylogenetic signal (Pagel’s *λ* and Bloomberg’s *K*). Overall, our study presents a methodological framework for identifying a reliable and standardized ramping rate to measure CT_max_ in ants, which can be applied to other ectotherms. Particular attention should be given to CT_max_ values obtained with less suitable ramping rates, and the potential biases they may introduce to trait-based research on global warming and habitat conversion, as well as inferences about phylogenetic conservatism.

## 1. Introduction

Organisms are increasingly exposed to novel and warmer environmental conditions owing to global changes such as deforestation, urbanization, and climate change. High temperatures, in particular, limit species’ survival, reproduction, and foraging activities—especially for ectothermic organisms [1]. Therefore, measuring the upper thermal limits of ectotherms is key to obtaining valuable information needed for forecasting changes in community composition and species distributions in response to rising temperatures [2].

Thermal performance theory [3] represents a useful framework for describing an organism’s performance in function of the temperature experienced [4], and for delimiting the thermal range within which an organism can remain active. The upper thermal maximum, also termed the Critical Thermal maximum (CT_max_), is a particularly important threshold that represents the temperature at which an organism is unable to withstand heat stress [5]. Investigating upper thermal limit is paramount for understanding how species’ fitness are impacted by climate change [6]. However, it is challenging to forecast the impacts of rising temperatures on species’ fitness due to the lack of standardized methods for measuring CT_max_ as well as their incompatibility with field observations [7]. For instance, the ramping rate (i.e., the rate at which temperature increases over time) used to measure CT_max_ varies substantially across studies, and this can result in major differences in CT_max_ values for the same species [5, 7, 8]. Therefore, a biologically relevant and reliable method for measuring CT_max_ values that is directly applicable to ecological research is urgently needed to provide meaningful estimates of species’ maximal thermal limits.

CT_max_ was defined by Cowles and Bogert as “*the thermal point at which locomotory activity becomes disorganized and the animal loses its ability to escape from conditions that will promptly lead to its death*” [9]. The measurement of CT_max_ uses an experimental approach to determine the upper thermal limit of an organism through a progressive increase of the environmental temperature (i.e., at the ramping rate) until the organism experiences a loss of muscle control or a heat-coma.

The use of ecological methodology should be standardized and comparative [10] to provide a consistent method for a given taxon or across multiple taxa. Yet, the use of the ramping rate has not been standardized across CT_max_ assays, resulting in problems when comparing CT_max_ values obtained from different ramping rates. Misinterpretations can also emerge in ecological studies that fail to consider this source of methodological bias [5, 7]. Although recent studies have developed a biophysical model based on *Drosophila* flies [11, 12], it should be noted that model exceptions are already known in other taxa such as ants [see 13 and S3 Appendix]. Additionally, it is crucial to select biologically relevant ramping rate(s) that will yield CT_max_ values that best reflect a species’ functional thermal niche from the perspective of community and functional ecology. Ultimately, the current lack of standardization and testing for ecological relevance leaves the following question unresolved: how should one develop a framework to test the reliability and ecological implications of the ramping rate used in the measurement of CT_max_?

In the present study, we use dynamic and static thermal assays, as well as field observations to capture different aspects of the thermal tolerance of ants—model organisms for understanding the ecophysiology of terrestrial ectotherms [5, 14, 15]—to investigate the correspondence and biological relevance of experimental measurements of CT_max_. Integrating both dynamic and static thermal assays allows us to fully capture the thermal tolerance of an organism, which depends on two main parameters: 1) the temperature to which the organism is exposed, and 2) the duration for which the organism is exposed to the given temperature [11]. To provide a general CT_max_ framework for studying the upper thermal limits of different taxa, we investigate the upper thermal limits of 27 ant species displaying different body sizes and which are associated with different micro-habitats, phylogenetic clades, and biogeographic origins. Our goals are to provide an overview of the limitations arising from the use of different ramping rates, and to identify a more reliable protocol for measuring biologically relevant and comparable CT_max_ values.

**Fig 1:**
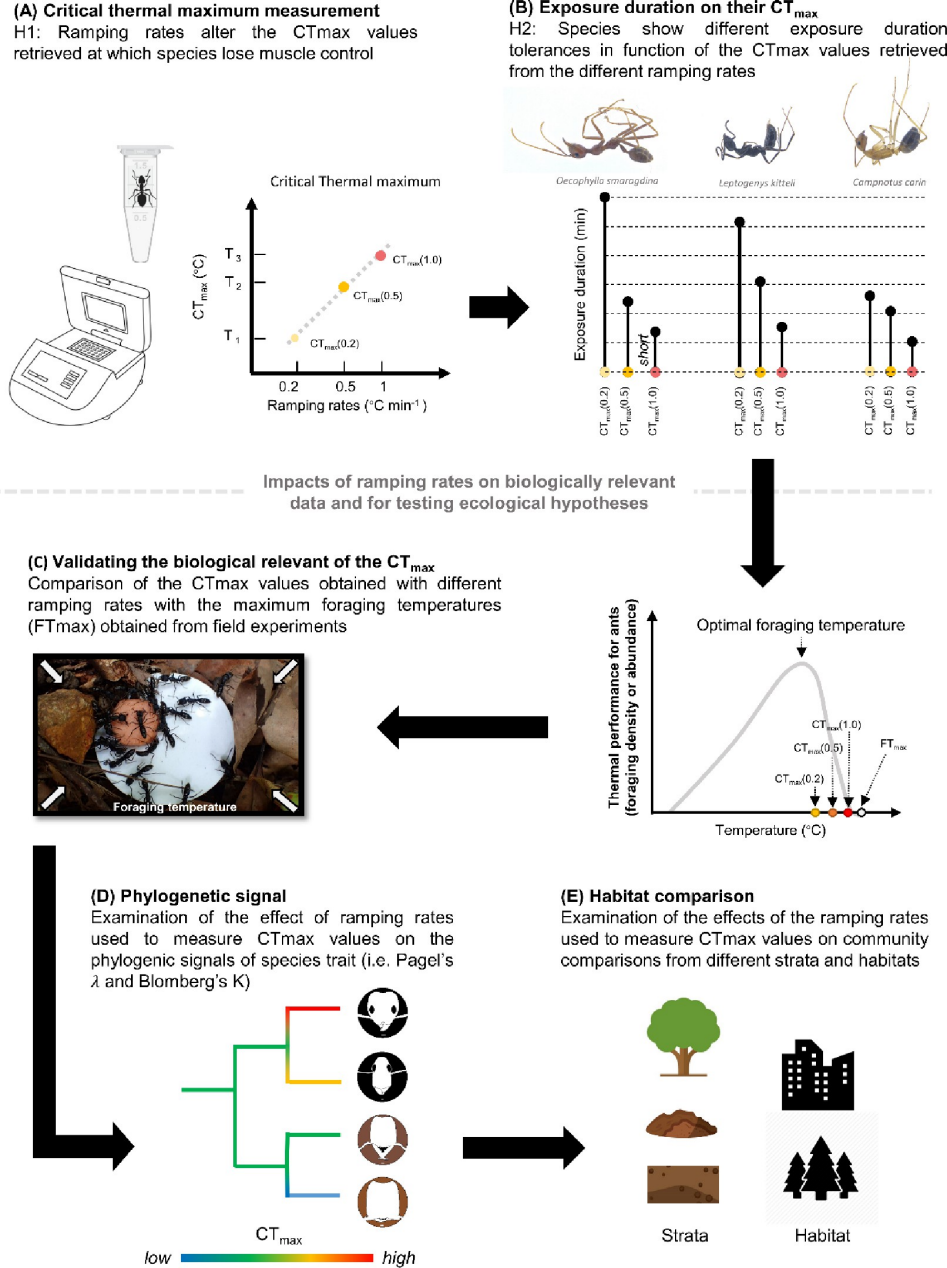
Study diagram of testing implications of ramping rates in the measurement of Critical Thermal maximum. **(A)** The first hypothesis (H1) examines if a positive relationship between the ramping rates and CT_max_ values exists for each ant species at the intraspecific level. **(B)** The second hypothesis (H2) examines the interspecific variations of exposure duration-based tolerance in function of the temperature treatments, we hypothesize that species assemblages show different exposure durations in their CT_max (0.2)_ values but presenting consistence in their CT_max (0.5)_ and CT_max (1)_ values. **(C)** Thermal performance framework of ectotherm on the basis of foraging behavior illustrate species activity in function of the temperature increase, FT_max_ recorded in the field presents critical and act as a thermal threshold for the organisms; the thermal performance curve is predicted based on the ant foraging activity in function of temperature. Through the comparison between CT_max_ and FT_max_, the results can examine will the species stop at their CT_max_ if the environmental temperature reached their CT_max_ and provide a biologically relevant ranking of the CT_max_ values retrieved by different ramping rates (0.2, 0.5 and 1°Cmin^-1^). **(D)** Examination of the effect of ramping rates used to measure CT_max_ values on the phylogenic signals using *Pagel’s λ* and *Blomberg’s K*. **(E)** Examination of the effects of the ramping rates used to measure CT_max_ values on community comparisons from different strata and habitats.

First, we conduct a literature review to identify the different ramping rates that have been used to measure the upper thermal limits of ants, and to select the most commonly used rates (i.e., 0.2, 0.5, and 1.0°C min^-1^) that we test in subsequent experiments (S1 Fig in S1 Appendix). Second, we investigate how the ramping rate used in a dynamic assay affects the CT_max_ value retrieved. Here, we predict strong positive correlations between ramping rates and CT_max_ values (Fig 1A) [7, 16]. Third, we further test the exposure duration-based thermal tolerance of most species, by using static assays to examine how species respond—in terms of their exposure duration—to the CT_max_ values retrieved from the dynamic essays at different ramping rates. Our literature review suggests that ant species tend to have relatively longer exposure duration-based tolerance at slow ramping rates (e.g., 0.2°C min^-1^) in comparison to faster ramping rates [e.g. 17–19]. We predict that for a given species, individuals exposed to the temperature of their CT_max_ retrieved at a low ramping rate (i.e., 0.2°C min^-1^) will show a more heterogenous and lengthier duration of tolerance, while those exposed to the temperature of their CT_max_ retrieved at a faster ramping rate (i.e., 0.5 and 1.0°C min^-1^) will display a shorter duration of tolerance. Fourth, we determine each species’ foraging temperature maximum (FT_max_) and compare this to their CT_max_ to investigate the concordance between laboratory- (i.e., CT_max_) and field-based (FT_max_) estimates of thermal tolerance. The FT_max_ represents the upper thermal limit of an ant species under natural conditions, and corresponds to a species’ thermal threshold prior to the loss of muscle control. To examine whether ant species in the field cease their foraging activities at the CT_max_ values retrieved with slow or fast ramping rates, we compare their CT_max_ values retrieved from dynamic assays in three different treatments (each with a different ramping rate) with the maximum temperature at which their activity was observed (i.e., FT_max_) during year-long field observations (Fig 1C). In theory, CT_max_ represents the maximum temperature to which an individual of a species can be exposed before it loses muscle control. Thus, comparing ant species’ CT_max_ values with their foraging performance in function of the temperature in the field (FT_max_) will allow us to test the ecological relevance of the CT_max_ values retrieved from different ramping rates. Finally, we investigate how the use of specific ramping rates can alter conclusions about the detection of phylogenetic signal (Fig 1D) as well as differences in species’ use of habitats and microhabitats (Fig 1E) in empirical studies comparing multiple species.

## 2. Materials and methods

### 2.1 Literature collection

We identified published studies of ant species’ upper thermal limits and recorded the relevant information describing the protocols that were used to measure CT_max_ in each study. This information included: whether a dynamic and/or static assay was used, the starting temperature, ramping rate, and duration of exposure. We conducted the literature search in Google Scholar (Google, USA), using one or combinations of the following keywords: “ant,” “CT_max_,” “Critical Thermal Maximum,” “Formicidae,” “ramping rate,” “thermal tolerance,” and “thermal limit”. All articles published from January 1944 to June 2020 were considered. In addition, we searched the CT_max_ database, GlobTherm [20], to obtain the methodological details (e.g., year, study species, locality, ramping rate, starting temperature) corresponding to each study.

### 2.2. Sampling ants for thermal tolerance measurements

We sampled a diversity of ant species with the goal to test whether the results retrieved could be generalized to a majority of taxa or limited to a particular subset of species. Several factors have shown to correspond to variation in upper thermal limits within a taxon [14]; four of these were used to guide our selection of ant species (S1 Table in S1 Appendix). First, we targeted ants from different microhabitats (i.e., subterranean ants, above-ground foraging ants, and arboreal ants) to account for the important differences in the magnitudes and fluctuations in temperature experienced by organisms living in different microhabitats, which vary in their sun exposure and buffering effects (e.g., from the canopy or soil), and which thus correspond to different thermal niches in the ecosystem [21]. The thermal tolerances of ant species are known to correlate with the fluctuations and extremes in temperatures observed in these microhabitats [22, 23]. Similarly, we included species collected from urban areas and forests to account for the wide range of conditions to which ants may be exposed across the thermal landscape. Second, we targeted ant species displaying substantially different individual body sizes (S1 Table in S1 Appendix) to account for the effect of size on thermal tolerance, which results from thermal inertia [24] and heat dissipation [25]; and also because size is known to affect other physiological processes in ants, such as desiccation [26]. Third, we targeted species from different phylogenetic clades to represent a range of species with different evolutionary histories for ultimately testing the impact of different CT_max_ treatments on the results retrieved (see below). The importance of phylogenetic constraints on determining upper thermal limits in ectotherms is debated, with studies presenting contradicting results [6, 27, 28]. Finally, biogeographic origin provides a wider range of natural history and microclimate with species evolving under different constraints (e.g., the natural barrier of temperature may be stronger in tropical environments) [29]. To ensure that the trends retrieved do not represent local adaptations, our sampling included several exotic species whose native range and thus evolutionary center differed from local species.

For this study, 27 ant species were collected in the field (S2 Table in S1 Appendix). The sampling was conducted in both secondary forests and urban habitats in Hong Kong SAR (22.3193°N, 114.1694°E) and Macao SAR (22.1987°N, 113.5439°E), China, during the dry season of 2018 and the wet season of 2019 (characterized by a tropical monsoonal climate). Research and collecting permits were obtained from the local governments of Hong Kong SAR and Macao SAR, and no protected species were sampled. Ant colonies were collected by hand, using an insect aspirator. Whenever possible, three hundred individuals were collected. Dynamic and static assays of thermal tolerance were conducted at least two hours after the colonies were transported from the field to the laboratory, but not more than 72 hours so as to prevent the individuals from acclimating to laboratory conditions under longer periods, which could modify the CT_max_ values measured [30]. For species with small colonies, the maximum number of workers available were collected; the individuals were later separated into groups of equal size and allocated to the different treatments, as far as possible including 15 individuals (minor workers in polymorphic species) in each treatment. We also included multiple colonies of *Crematogaster rogenhoferi* (N = 2) and *Solenopsis invicta* (N = 3) to examine the effects of different ramping rates on their CT_max_ in dynamic assays and on their exposure duration-based tolerance in static assays. Upon collection, all individuals were transported to the laboratory (24 ± 2°C; 57.5 ± 5% relative humidity) for CT_max_ assays, and provided with wet cotton. The different treatments in each assay were run sequentially to limit the effects of acclimation.

### 2.3 Dynamic assays (continuous changes in temperature to determine thermal limits)

To experimentally quantify the upper thermal limits of the ants, we conducted dynamic assays of their Critical Thermal maximum (CT_max_) based on a continuous increase in temperature over time. Specifically, we placed individual ants in an environment in which the temperature was increased progressively and steadily according to the predefined ramping rate (°C per minute). We used a general protocol for measuring CT_max_ in ants adapted from [31], with three different ramping rates set as the experimental treatments (see below and S2 Fig in S1 Appendix). We measured the CT_max_ of 27 species with different microhabitat preferences (i.e., associations with different vertical habitat and strata) using a digital dry bath (Benchmark—BSH1004, advertised accuracy ± 0.2°C). For most species, 15 individuals were tested in each treatment (i.e., a total of 45 individuals per species, S3 Table in S1 Appendix); each replicate comprised an individual ant worker placed within a 2.0 mL Eppendorf tube, with its cap filled with cotton to prevent individuals from taking refuge at this location. In addition, to limit the stress experienced by individual ants and the release of defensive chemicals (e.g., formic acid) which in the closed environment could be harmful, we guided the ants into their respective Eppendorf tubes instead of picking them up with forceps. Each individual was used only once, as a repeated exposure to high temperatures in multiple assays could cause heat injury, resulting in lower CT_max_ values. To ensure that the temperature recorded as the CT_max_ was the indeed the temperature experienced by the individual, an extra digital thermometer (UEi Test Instruments DT302 Dual Input IP67) with its sensor placed inside a supplementary Eppendorf tube (that was also in the dry bath) was used as a temperature control; the reading from this thermometer represented the most accurate temperature corresponding to the loss of muscle control.

Ramping rates of 0.2, 0.5, and 1.0°C min^-1^ were selected based on a systematic review of previous studies (see 2.1 literature collection and the list in S2 Appendix); these also reflect environmental fluctuations observed within terrestrial ecosystems [21, 32]. The small bodysizes of ants make them ideal model organisms for tracking the effects of changes in environmental temperature on body temperature. For instance, a study conducting a similar CT_max_ assay showed that an ant’s body temperature tracked the temperature of the inner surface of the Eppendorf tube in which it was placed, as ant stood on the surface and had limited heat buffering abilities [14]. To account for the similarity between the environmental and experienced temperatures by the individual, we also conducted a preliminary study using an infrared thermal camera to measure the body-surface temperature of ants, as this would allow us to infer their body temperatures [33]. This experiment showed that emissions of environmental heat are easily transferred to individual ants, with their body-surface temperatures increasing by up to 9–10°C within one minute. It thus indicated that ant bodies possessed limited heat inertia in our thermal assays (S3 Fig in S1 Appendix), and could easily track the highest ramping rate we tested (i.e., 1°C per minute).

The starting temperature of each dynamic assay was set at 36°C, a common starting temperature for CT_max_ assays in ants [34]. In keeping with the protocols used in previous CT_max_ studies for ants [e.g. 18, 35], the individuals were directly exposed to the experimental temperature without being subjected to long periods of acclimation in the laboratory. Depending on the treatment tested, we gradually increased the temperature at either 0.2, 0.5, or 1.0°C min^-1^ (Fig 1), until the individual was observed to display a loss of muscle control (i.e., the onset of spasms), and the corresponding temperature was recorded as the CT_max_ value of that individual. The loss of muscle control was defined as the thermal limit of the individual, because it is more relevant to biological survival than the lethal temperature [36].

### 2.4 Static assays (exposure duration-based tolerance at constant temperature)

Here, a static assay refers to the experimental use of a constant temperature to measure an individual’s thermal tolerance in terms of the duration for which it can withstand being exposed to that temperature until it experiences a loss of muscle control. It should be noted, however, this exposure duration-based definition of CT_max_ has not been considered in the previous studies [e.g., 5]. We used static assays to investigate species’ exposure duration-based tolerances in function of their different CT_max_ values retrieved from the dynamic assays using the three different ramping rates. Ant workers from the same colony were first used in the dynamic assays (see above), while other individuals from the same colony were used for the static assays. This experimental order eliminated inter-colony variation in thermal tolerance measurements of static and dynamic assays. Three of the 27 species tested were not used in the static assays due to the limited number of individuals available.

In the static assay, we placed an individual ant in an environment with a fixed temperature and measured the duration for which it could tolerate that condition. The experimental temperatures used for the static assays were determined from the CT_max_ values obtained in the dynamic assays at different ramping rates (0.2, 0.5 or 1.0°C min^-1^), such each species was measured in three separate static assays (treatments), each using a different temperature (i.e., CT_max(0.2)_, CT_max(0.5)_, or CT_max(1)_) that was specific to that species (Fig 1). For most species, we used 15 individuals in each of the three treatments and another 15 individuals as a control group (S4 Table in S1 Appendix). We used the same experimental setup and the same procedure to record the loss of muscle control as those used in the dynamic assays. In the static assays, the temperature remained unchanged, and we checked for each individual’s loss muscle control in one-minute intervals over a maximum duration of 30 minutes. A period of 30 minutes represents a relatively long duration, which in natural conditions should allow sufficient time for an individual to locate a thermal refuge, thus avoiding exposure to its upper thermal limit. For instance, individuals of the desert ant, *Cataglyphis bombycina*, can only tolerate their upper thermal limits for about 10 minutes in the field [37, 38].

### 2.5 Estimating foraging temperatures

From the perspective of niche theory, the foraging temperature represents the realized niche—the range of temperatures at which a given species can be active at in the field—while CT_max_ is considered the fundamental niche of a species’ physiological response to temperature. Ants are social insects living within a nest (a climatic refuge), and thus make decisions on whether to forage based on various factors, including temperature [39], with many species presenting recruitment behaviors. As such, ants are model species for studying the behavioral responses of animals to changes in temperature [37]. In order to collect data on the temperatures at which ants forage, we used baits to observe the occurrences and recruitment patterns for a wide range of species during the day. Each baiting station was composed of a white disk (Ø 4.7cm) laid on the ground surface and on which a 4 mm slice of sausage (®Valley Chef) was deposited in its centre. The sausage was used as its composition includes proteins, lipids and carbohydrates, which are attractive to numerous species; moreover, the calibrated and circular size provides a standardized amount of food available to the species between replicates. Species’ foraging temperatures were recorded as the ground-surface temperatures at which they were observed to recruit to the baits, and were measured using an infrared thermometer (Fluke 62 MAX+) from one meter above the ground. We took the average of temperatures measured at four cardinal locations from a distance of 2 to 5 cm from the edges of the white disk (S5 Fig in S1 Appendix). Ground-surface temperatures are the most relevant environmental temperatures experienced by ants; with maximum foraging temperature representing the upper thermal limit observed in the field [40].

From 2015 to 2018, > 11817 baits were installed in different localities in Hong Kong from 1000–1600 HRS, which corresponds to the warmest period of the day. The baits were positioned along transects, with a minimum distance of 10 m separating two baits. Baiting was conducted throughout each year, and baits were placed predominantly within open urban and suburban environments. The baits were left to operate for a period of 1 to 2 hours, and the ant activity at each bait was noted every 15 minutes. Ant foraging activity was noted on 10,157 baits. We used the foraging temperature maximum of seven dominant species (*Anoplolepis gracilipes*, *Monomorium chinense*, *Paratrechina longicornis*, *Pheidole megacephala*, *P*. *parva*, *Solenopsis invicta* and *Tapinoma melanocephalum*), which each had at least 430 records (max. = 1764). These seven species were observed at a total of 7,692 baits across multiple seasons in Hong Kong. These species were selected on the basis of the large number of records available, and their abundances in open habitats, where ground-surface temperatures can be very high (the maximum value recorded during our study was 66.2°C).

### 2.6 Statistical analyses

#### 2.6.1 Ramping rates

First, individual CT_max_ was averaged by species identity and ramping rate. To assess the importance of the ramping rate in CT_max_ measurements, we used a linear mixed model, with species-average CT_max_ as the response, and ramping rate as the sole predictor. To control for phylogenetic dependence, we specified species nested within genus and subfamily as random intercepts. Second, we performed a Bartlett’s test to assess whether the 24 ant species exhibited more heterogenous exposure duration-based tolerances of temperatures corresponding to their CT_max_ values measured at a low ramping rate in the static assays. Thirdly, we performed ANOVA and Tukey’s tests to assess if increasing the ramping rate led to a higher observed CT_max_ for each species; and preformed Kruskal-Wallis and Dunn’s tests to assess whether each species had a shorter exposure duration-based tolerance of the temperature corresponding to its CT_max_ that was measured using a fast ramping rate. Finally, we performed 27 simple linear models for dynamic assays to examine the CT_max_ impacted by the ramping rate in the supporting information, and reported the adjusted R^2^ values in explaining intraspecific variations within the dataset (see S5 Table in S1 Appendix).

#### 2.6.2 Critical Thermal maximum vs. foraging temperature maximum

For comparing CT_max_ and FT_max_ in each species, we used their absolute difference and calculated the mean and standard deviation for each ramping rate (FT_max_ of each species was defined as the maximum foraging temperature observed across all the foraging temperatures recorded). In addition, comparisons between CT_max_ and the top 1% of FTs was also conducted to account for the variation in foraging temperature maximum that can be observed within different ant populations. We also used a linear model with the ramping rate and species identity as the predictors and the absolute differences as the response to examine how both factors (i.e., ramping rate and interspecific variation) affected species’ CT_max_ and FT_max_. Because we only had sufficient foraging records (N > 500) for seven species, and these species generally represented different genera and subfamilies, we used a simple linear model for the comparative analyses.

#### 2.6.3 Phylogenetic signal analyses

To test if the choice of ramping rate would affect the results of a phylogenetic analysis, we generated a genus-level phylogeny comprising the genera of our study species. Here, we used a backbone tree from a published genus-level phylogeny [41] and applied tree pruning to keep a single species for every genus. Then we simulated 1,000 species-level phylogenies using a Yule (pure-birth) process with the function *genus*.*to*.*species*.*tree* in the Rpackage “phytools” (Revell 2012). For each species-level phylogeny, we used *Pagel’s λ* and *Blomberg’s K* to examine the phylogenetic signals in CT_max_ generated from the different treatments (i.e., ramping rates). We also tested whether *λ* and *K* were significantly different from random using a likelihood ratio test and a randomization test (1,000 randomizations), respectively.

#### 2.6.4 Habitat and microhabitat comparisons

Ant species experience different variations in temperature based on the habitats and microhabitats in which they live [14, 35]. For habitat and microhabitat comparisons of species’ CT_max_, we again used linear mixed models to analyze how a change in the ramping rate used could alter conclusions about the CT_max_ of species from different habitats. We built one model to compare species from different vertical strata (i.e., subterranean ants, above-ground foraging ants, and arboreal ants) and another to compare those from different habitats (i.e., urban and forest). Each model included CT_max_ as the response, and ramping rate, strata (or habitat), and their interaction as the predictors. We included species nested within genus and subfamily as random intercepts. We further conducted pairwise comparisons between strata or habitats within the same ramping rate and obtained the Tukey-adjusted *p*-value, which indicated the detection of a significant interaction. Finally, we assessed whether habitat/strata differences in CT_max_ varied with the use of different ramping rates by setting the null value as the weakest effect size (instead of zero).

All statistical analyses were performed in R version 3.6.2 [42]. Bartlett’s test of homogeneity of variances was conducted using the function *bartlett*.*test*. Linear models were performed using the function *lm*. Linear mixed models were performed using *lme4* and *lmerTest*, with Tukey’s post-hoc comparisons performed using *emmeans* [43]. White-adjusted *p*-values were obtained using the function *Anova* in R-package “car” [44] to control for the effect of a variance in heteroscedasticity.

## 3. Results

### 3.1 Literature review of studies on ant species’ upper thermal limits

We retrieved a total of 51 publications (49 studies using dynamic assays and two using static assays) investigating ant species’ upper thermal limits between January 1944 to June 2020 (see S2 Appendix). In total, 20 different values of ramping rate were used, with 0.2°C min^-1^ (13/49; 27%) and 1.0°C min^-1^ (22/49; 45%) being the most widely used ramping rates (S1 Fig in S1 Appendix).

### 3.2 Dynamic assays (Critical Thermal maximum CT_max_)

**Fig 2:**
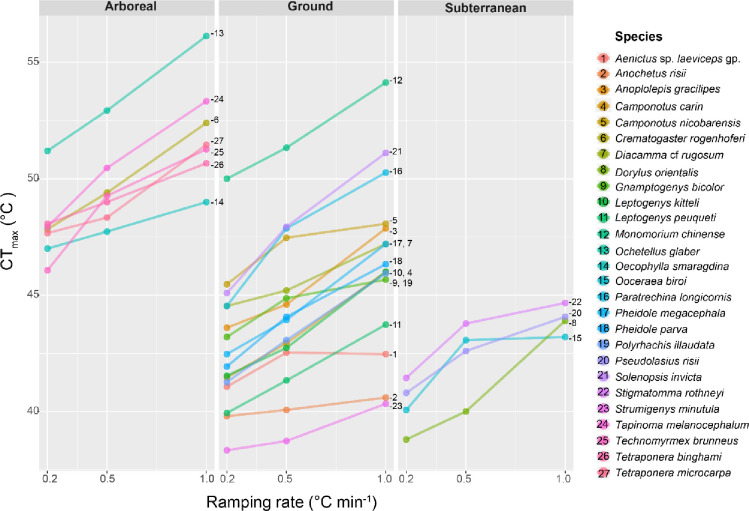
Results of dynamic assays. Line plots of CT_max_ values measured in function of three ramping rates used for 27 ant species found in function of their vertical stratification (arboreal, ground and subterranean strata).

A total of 1,743 individuals from 27 species were used for dynamic assays. In the intraspecific models, the CT_max_ values retrieved were dependent on the ramping rate used; with fast ramping rates resulting in significantly higher CT_max_ values than slow ramping rates for all species (*p*-value < 0.001, Fig 2, S3 Table in S1 Appendix). Differences in the CT_max_ values retrieved between the slow ramping rate (i.e., 0.2°C min^-1^) and the fast ramping rate (i.e., 1°C min^-1^) averaged 4.13°C, ranging from 1.40°C in *Aenictus* sp. *laeviceps* gp. to 6.47°C in *Crematogaster rogenhoferi* (S3 Table in S1 Appendix). The CT_max_ values for most (i.e., 26 out of 27) species were correlated with the ramping rates used in the intraspecific models (adjusted R^2^ = 0.629 [0.229–0.95], *p*-value < 0.05; S5 Table in S1 Appendix), with the exception of *Anochetus risii* (Adjusted R^2^ = 0.090, *p*-value = 0.052; S5 Table in S1 Appendix). Mixed models including data of all species revealed that CT_max_ values were positively correlated with ramping rate (Marginal R^2^ = 0.126, *p*-value < 0.001; Table 1). Species identity was a strong predictor of variation in CT_max_ values; including this variable as random effects in the linear mixed model led to a marked improvement in explanatory power (Conditional R^2^ = 0.942; Table 1).

**Table 1:** Outcomes of the linear mixed model examining the relationship between parameters (CT_max_, ramping rate and species identity as well as the genus and subfamily identity) by linear mixed model for the dynamic assay with white-adjusted *p*-value.

CT_max_ ~ Ramping rate + (1| Subfamily /Genus/ Species)					
	Chi square	DF	*p*-value		
Ramping rate	181.65	2	*<0*.*001*		
Marginal R^2^: 0.126 and Conditional R^2^: 0.942					
**Random effects**					
**Groups**		**Variance**	**Std. Dev.**		
Species: (Genus: Subfamily)	Intercept	1.707	1.307		
Genus: Subfamily	Intercept	7.702	2.775		
Subfamily	Intercept	6.542	2.558		
Residuals		1.122	1.059		
**Fixed effects**					
	**Estimate**	**Std. Error**	**df**	**t-value**	***p*-value**
Intercept	43.7481	1.1766	6.805	37.182	*<0*.*001*
Ramping rate– 0.5°C min^-1^	1.6325	0.2831	55.018	5.767	*<0*.*001*
Ramping rate– 1°C min^-1^	3.8025	0.2831	55.018	13.433	*<0*.*001*

### 3.3 Static assays (exposure duration-based tolerance to a constant temperature)

**Fig 3:**
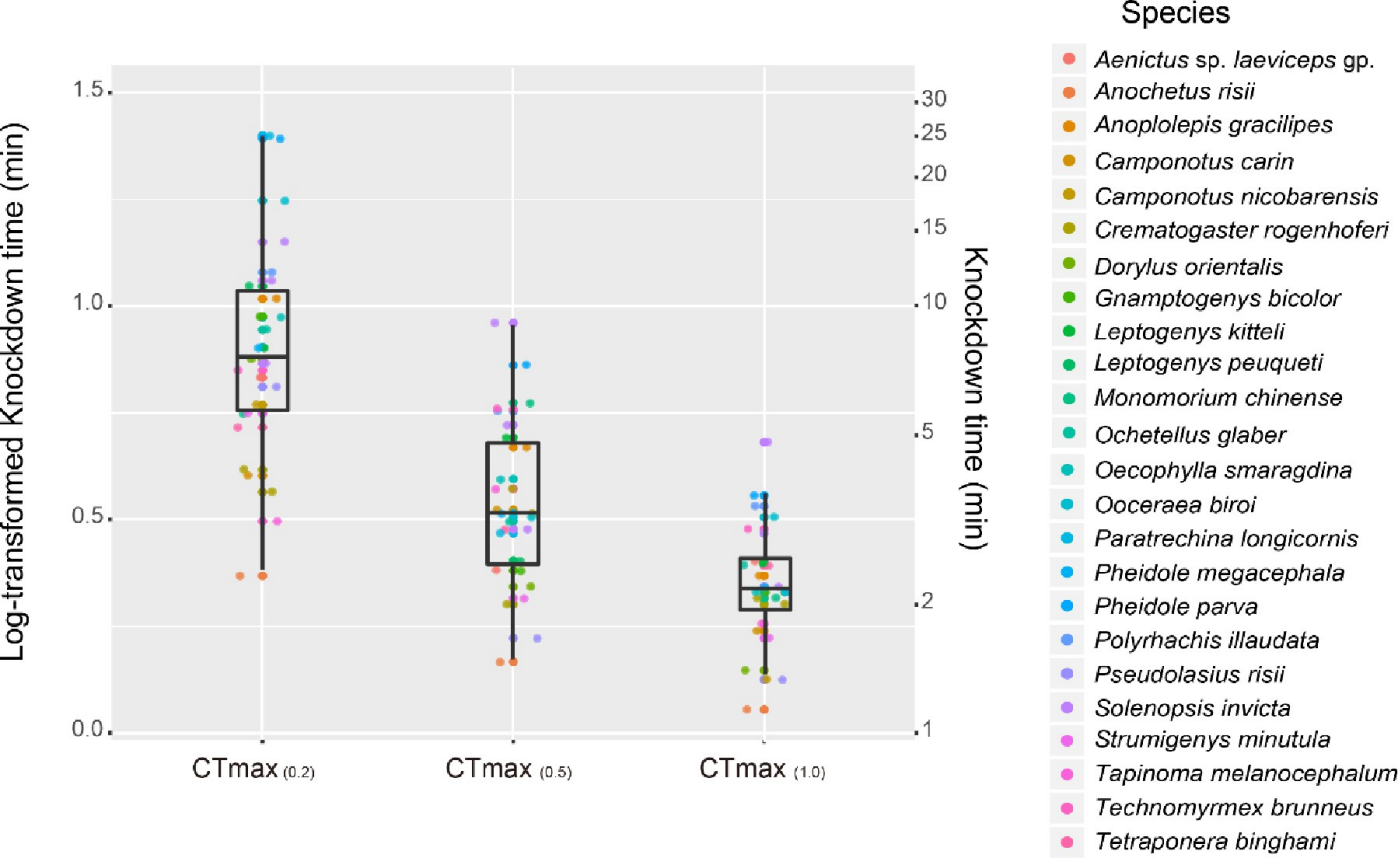
Results of static assays. Mean exposure duration-based tolerance values (±SE) of 24 ant species for three temperatures based on the values retrieved in the CT_max (0.2, 0.5, and 1.0)_ treatments. Right y-axis refers to the duration tolerance the ants were maintaining their muscle control, and left y-axis refers to the log-transformed duration tolerance values.

A total of 1,191 individuals from 24 species were used for static assays. The loss of muscle control was observed within 30 minutes in all individuals of the 24 species exposed to the temperatures corresponding to their CT_max(0.5)_ and CT_max(1.0)_. However, this was not the case in static assays for temperatures corresponding to species’ CT_max (0.2)_, where for four species, only 40–96% of the 15 individuals tested displayed this condition (S4 Table in S1 Appendix). The exposure duration-based tolerances of 24 species were affected by the specific ramping rates used to measure their CT_max_, with different ramping rates leading to significantly different exposure durations (*p*-value < 0.001, Kruskal-Wallis test and Dunn’s test). At CT_max(0.2)_, species’ exposure durations (i.e., the duration required to initiate a loss of muscle control) ranged from 2.3 min. (*A*. *risii*) to 17.8 min. (*Paratrechina longicornis*) (Mean ± SD: 8.5 ± 4.3 min); this represented the largest interspecific variation in exposure duration observed among the three temperature treatments tested (CT_max (0.2, 0.5 and 1.0)_, S4 Fig in S1 Appendix). At CT_max (0.5)_, species’ exposure durations ranged from 1.5 min. (*A*. *risii*) to 5.8 min. (*Solenopsis invicta*) (Mean ± SD: 3.6 ± 1.5 min). At CT_max (1.0)_, almost all species showed similar exposure durations (Mean ± SD: 2.2 ± 0.7 min), with the longest exposure duration being 3.6 min. (*Pheidole parva*) (S4 Table in S1 Appendix and Fig 3). For each static assay, the responses of the 24 species tested showed the smallest interspecific variation in terms of exposure tolerance duration (SD: 0.685 min.) for values retrieved from the CT_max (1.0)_ treatment. The 24 ant species displayed high interspecific variation in exposure duration in static assays for temperatures corresponding to their CT_max (0.5)_ and CT_max (0.2)_ (SD: 1.5 at CT_max (0.5) and_ SD: 4.3 at CT_max (0.2)_, S4 Table in S1 Appendix). Additionally, their exposure durations displayed unequal variances across the three static assays corresponding to their CT_max (1.0)_, CT_max (0.5)_ and CT_max (0.2)_ (*p*-value < 0.05 in the Bartlett’s test of homogeneity of variances, S4 Fig in S1 Appendix).

### 3.4 Foraging temperature maximum vs. Critical Thermal maximum

**Fig 4:**
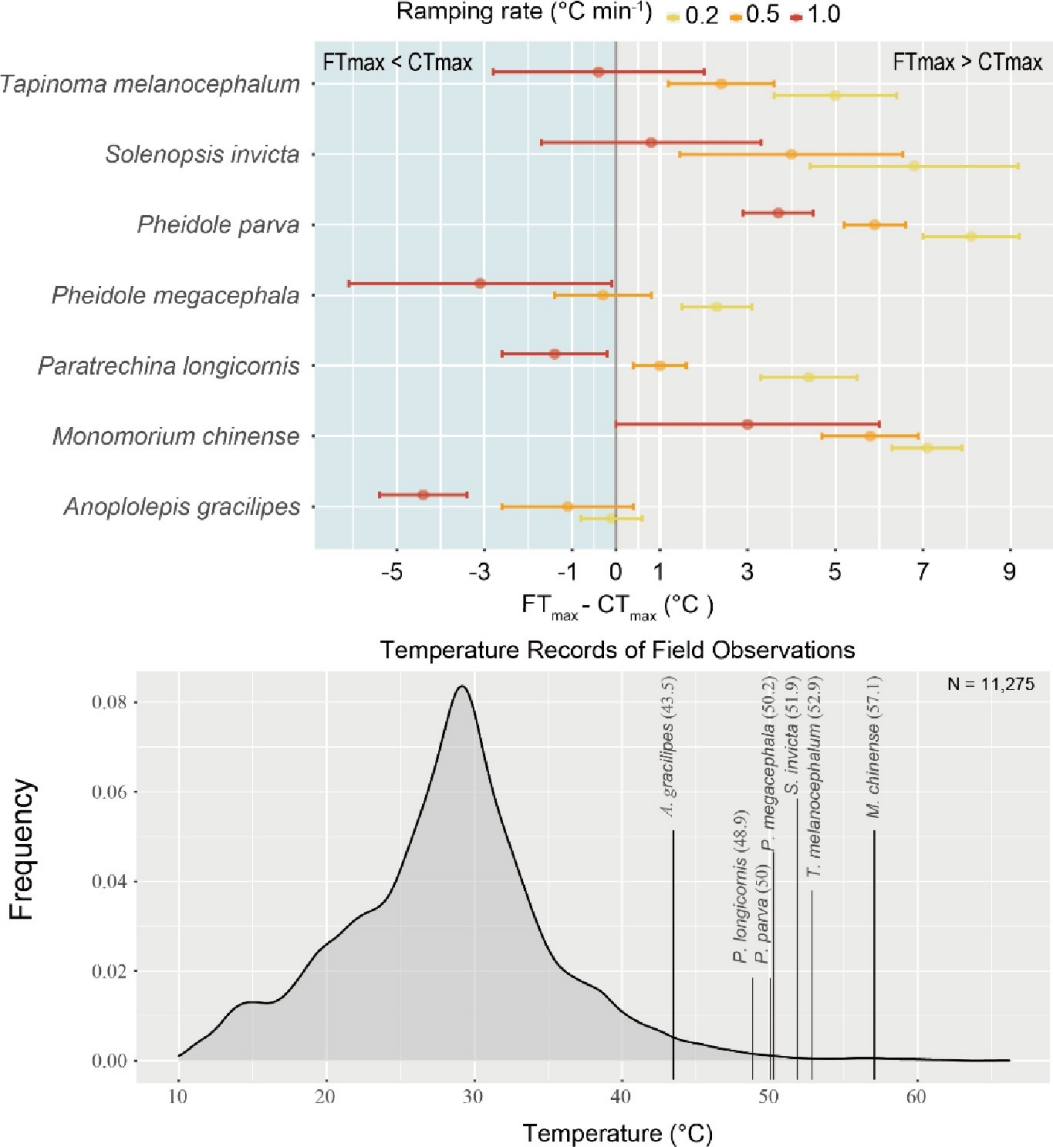
Critical Thermal maximum vs. foraging temperature records. Upper plot showing the difference between FT_max_ and CT_max (0.2, 0.5, and 1.0)_ and errors bars as standard deviation of the CT_max_ values. Lower plot shows the range and distribution frequency of all surface temperatures measured near baiting stations during the sampling period, independently of the presence of ants or not. Vertical lines indicate the FT_max_ values measured for each species in the field.

For five out of the seven species tested, CT_max (1.0)_ was the closest to their FT_max_ value measured (Fig 4) as well as within the top 1% of their FTs. Specifically, absolute differences between FT_max_ and CT_max_ values were lowest when a 1°C min^-1^ ramping rate was used to measure CT_max_ in the dynamic assay (Mean ± SD: CT_max (1.0)_: 2.39 ± 1.41; CT_max (0.5)_: 3.79 ± 2.13; CT_max (0.2)_: 5.60 ± 2.57, Fig 4 and Table 2); a similar trend was observed when FT_max_ was defined as the top 1% of a species’ FTs (Table 2). Overall, the comparison between FT_max_/1% top FT and CT_max_ values was significantly different between the three ramping rates (*p*-value < 0.05 and adjusted R^2^ = 0.489 for FT_max_; *p*-value < 0.05 and adjusted R^2^ = 0.471 for 1% top FT; Table 2), indicating a better overall performance in reconciling field and laboratory data.

**Table 2:** The comparsion between CT_max_ values retrevied from three different ramping rates and FT_max_ /1% top FT (sample size of each species shown on Fig 4), the lightly gray color background refers the CT_max_ value, closer to the FT_max_ for those species.

Species	| FT_max_—CT_max_ | (°C)		
	0.2°C min^-1^	0.5°C min^-1^	1°C min^-1^
*Anoplolepis gracilipes*	*0*.*1*	1.1	4.4
*Monomorium chinense*	7.1	5.8	*3*
*Paratrechina longicornis*	4.4	*1*	1.4
*Pheidole megacephala*	7.7	6.3	*3*
*Pheidole parva*	8.1	5.9	*3*.*7*
*Solenopsis invicta*	6.8	4	*0*.*8*
*Tapinoma melanocephalum*	5	2.4	*0*.*4*
**Mean of | FT**_**max**_**—CT**_**max**_ **| (°C):**	**5.60**	**3.79**	**2.39**
**Standard deviation**	**2.57**	**2.32**	**1.41**
| FTmax—CT_max_ | ~ Ramping rate + Species identity (*p*-value < 0.05, adjusted R^2^ = 0.4885)			
	**| 1% top FT—CT**_**max**_ **| (°C)**		
*Anoplolepis gracilipes*	7.1	5.8	*3*
*Monomorium chinense*	4.4	*1*	1.4
*Paratrechina longicornis*	7.7	6.3	*3*
*Pheidole megacephala*	8.1	5.9	*3*.*7*
*Pheidole parva*	6.8	4	*0*.*8*
*Solenopsis invicta*	*0*.*1*	1.1	4.4
*Tapinoma melanocephalum*	7.1	5.8	*3*
**Mean of | 1% top FT—CT**_**max**_ **| (°C):**	**5.90**	**4.27**	**2.76**
**Standard deviation**	**2.61**	**2.15**	**1.16**
| 1% top FT—CT_max_ | ~ Ramping rate + Species identity (*p*-value < 0.05, adjusted R^2^ = 0.4708)			

### 3.5 Phylogenetic signals

**Fig 5:**
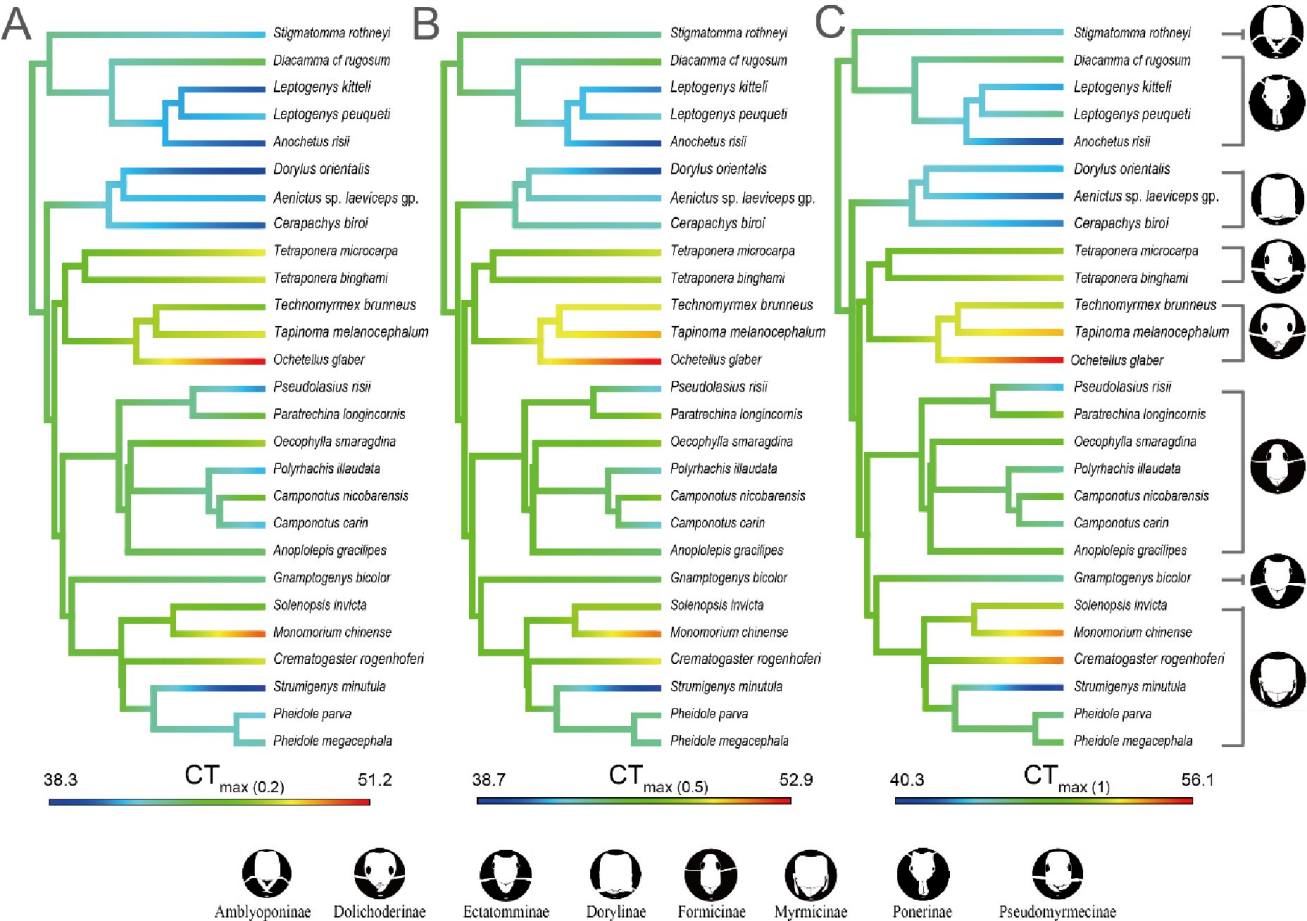
Critical Thermal maximum (CT_max_) of 27 ant species in function of the phylogeny. Color shading corresponds with the magnitude of thermal tolerance measured with different ramping rates, A. 0.2°C min^-1^, B. 0.5°C min^-1^, C. 1.0°C min^-1^. Ant illustrations credited to Mr Runxi Wang with permission.

Methodological approaches used for measuring CT_max_ resulted in differences in the significance of phylogenetic signals. Species’ CT_max_ values retrieved at 0.2, 0.5, and 1.0°C min^-1^ displayed different patterns of variation across the ant phylogeny (Fig 5). This effect of ramping rate on phylogenetic patterns was further confirmed in a genus-level polytomy tree and phylogenetic analyses. Specifically, CT_max_ displayed a stronger phylogenetic signal when measured at a ramping rate of 1.0°C min^-1^, as indicated by higher values of *Pagel’s λ* (Mean = 0.991) and *Blomberg’s* K (Mean = 0.906) (Table 3), but a weaker phylogenetic signal when the other ramping rates (i.e., 0.2 and 0.5°C min^-1^) were used for measurement, as indicated by the lower values of *Pagel’s λ* (0.827–0.862) and *Blomberg’s* K (0.768–0.781) (Table 3). In addition, the proportion of simulated trees that detected significant phylogenetic signals increased when a higher ramping rate was used to measure CT_max_ (S6 Fig in S1 Appendix).

**Table 3:** Phylogentic signals, *Pagel’s λ* and *Blomberg’s* K, for dynamic assay 0.2°C min^-1^, 0.5°C min^-1^, 1.0°C min^-1^ (1000 times).

		0.2°C min^-1^	0.5°C min^-1^	1.0°C min^-1^
*Pagel’s* lambda	Pagel’s λ:	0.700–1.151 (Mean: 0.862)	0.669–1.139 (Mean: 0.827)	0.879–1.191 (Mean: 0.991)
	*p* value:	0.0013–0.0644	0.0008–0.0521	0.0017–0.0215
	*p* value < 0.05:	96.30%	99.60%	100%
	*p* value < 0.01:	7.20%	8.90%	42.10%
*Blomberg’s K*	Blomberg’s K:	0.002–0.980 (Mean: 0.781)	0.002–0.965 (Mean: 0.768)	0.014–1.023 (Mean: 0.906)
	*p* value:	0.0003–0.5588	0.0001–0.5768	0.0004–0.2678
	*p* value < 0.05:	75.50%	72.60%	94.10%
	*p* value < 0.01:	31.20%	31.70%	85.80%

### 3.6 Habitat and microhabitat comparisons

The measured CT_max_ values of ant species were important for distinguishing their ecology. For instance, for all three ramping rates, the CT_max_ values of ant species occupying the subterranean, ground, and arboreal strata were significantly different (*p*-value < 0.01; Table 4). Specifically, arboreal species showed consistently higher thermal tolerances than ground and subterranean species (*p*-value < 0.05; Table 4). The ramping rate used did not influence the relationships between the CT_max_ values of species from different vertical strata, as no significant interaction between strata and ramping rate was detected (*p*-value = 0.559). There was, however, a significant interaction between habitat and ramping rate; CT_max_ values of species collected within urban habitats (N = 9) were significantly higher than those of species collected in forested habitats (N = 18, *p*-value < 0.001) for all three ramping rates tested. Nevertheless, we found that when a ramping rate of 1.0°C min^-1^ was used, the effect size of a difference in habitat on species’ CT_max_ (i.e., 5.16°C) was significantly larger than that observed when a ramping rate of 0.2°C min^-1^ was used (i.e., 3.42°C) (*p*-value = 0.003), which in turn was marginally different (*p*-value = 0.07) from the effect observed when a 0.5°C min^-1^ ramping rate was used (i.e., 4.34°C).

**Table 4:** Comparison of species CT_max_ values from habitats and microhabitats respectively, the linear mixed models examine the relationship between parameters (CT_max_, ramping rate and species identity) by linear mixed model for the dynamic assay with white-adjusted *p*-value; with species identify nested within genus and subfamily identity.

CT_max_ ~ Strata + Ramping rate + (1| Subfamily/ Genus/ Species)					
	**Chi square**	**Df**	***p*-value**		
Intercept	1609.786	**1**	*<0*.*001*		
Strata	18.021	2	*<0*.*001*		
Ramping rate	60.72	2	*<0*.*001*		
Strata: Ramping rate	3.018	4	0.555		
**Ramping rate 0.2°C min** ^**-1**^	**Estimate**	**SE**	**Df**	***p*-value**	
A.–G	5.465	1.55	18.6	*0*.*0062*	
A.–S.	7.269	2.24	23.6	*0*.*0093*	
G.–S.	1.804	2	24.6	0.6454	
**Ramping rate 0.5°C min** ^**-1**^	**Estimate**	**SE**	**Df**	***p*-value**	
A.–G	5.573	1.55	18.6	*0*.*0053*	
A.–S.	6.508	2.24	23.6	*0*.*024*	
G.–S.	0.935	2	24.6	0.8875	
**Ramping rate 1°C min** ^**-1**^	**Estimate**	**SE**	**Df**	***p*-value**	
A.–G	5.845	1.55	18.6	*0*.*036*	
A.–S.	8.194	2.24	23.6	*0*.*034*	
G.–S.	2.349	2	24.6	1.172	
**CT**_**max**_ **~ Habitat + Ramping rate + (1| Subfamily/ Genus/ Species)**					
	**Chi square**	**Df**	***p*-value**		
Intercept	2352.643	1	<0.001		
Habitat	32.831	1	<0.001		
Ramping rate	216.135	2	<0.001		
Habitat: Ramping rate	16.933	2	<0.001		
**Ramping rate 0.2°C min** ^**-1**^	**Estimate**	**SE**	**Df**	**Null**	***p*-value**
Forest—Urban	3.42	0.611	63.2	3.42	0.5012
**Ramping rate 0.5°C min** ^**-1**^	**Estimate**	**SE**	**Df**	**Null**	***p*-value**
Forest—Urban	4.34	0.611	63.2	3.42	0.0691
**Ramping rate 1°C min** ^**-1**^	**Estimate**	**SE**	**Df**	**Null**	***p*-value**
Forest—Urban	5.16	0.611	63.2	3.42	0.003

For the strata, A. = Arboreal, G. = Ground, S. = Subterranean.

## 4. Discussion

There has been an increasing interest the forecasting of species’ tolerances to warming environments based on their CT_max_ [19, 45]. Although design of ramping rate has been discussed since the development of CT_max_ [5, 7, 20], there is no consensus on what constitutes a suitable ramping rate, and arguments for slow as well as fast ramping rates have been made from various ecological, physiological and methodological aspects [5, 7, 8, 11, 46]. Here, using a combination of dynamic and static assays on a wide range of ant species with distinct ecological, morphological, phylogenetic and biogeographic characteristics, our results evidence a consistent trend between the ramping rate used and the CT_max_ values retrieved, suggesting that a major part of the variation observed in (and among) species’ CT_max_ values results from differences in the methodological approaches that have been used [5, 7, 11]. Furthermore, we propose that CT_max_ values retrieved from a fast ramping rate (1.0°C min^-1^) are the most biologically relevant, and evidence this using an additional experimental approach (i.e., static assays) as well as field observations of species’ FT_max_ values. Overall, our study provides important experimental and field-based evidence to guide the selection of a reliable ramping rate for CT_max_ measurements of ant species. This approach may also be applied to numerous other terrestrial ectotherms.

### 4.1 Literature review of Critical Thermal maximum in ants

In reviewing the literature on ant species’ upper thermal limits, we find that an overwhelming number of studies (49/51) have used the dynamic assay approach, confirming previous observations of studies across a wide range of taxa [5]. Our review also shows that an extensive variety of ramping rates (0.05–2.0°C min^-1^) have been used to measure ant species’ CT_max_. Furthermore, the two most frequently used ramping rates—0.2 and 1.0°C min^-1^—exhibit a fivefold difference in magnitude (S1 Fig in S1 Appendix). One emerging issue with such methodological differences among studies is that the results obtained from assessments using different ramping rates are not directly comparable [10]. This also implies that unless CT_max_ values are somehow corrected for ramping rate, the conclusions of meta-analyses may be unreliable, as the trends observed are likely to be strongly impaired by methodological artifacts.

### 4.2 Use of ramping rate in CT_max_ assay

Since the implementation of CT_max_ measurements, there has been much debate over the ramping rate used, as well as the tradeoffs between the use of slow versus fast ramping rates [5, 20, 46]. The use of different ramping rates to measure species CT_max_ is controversial and has not been standardized [17]. Across studies of ant species’ thermal tolerances, the most frequently used ramping rates have been 0.2, 0.5, and 1°C min^-1^ (S1 Fig in S1 Appendix). Among these, the results from our experiments on 27 ant species clearly support the use of the faster ramping rates (i.e., 0.5 and 1°C min^-1^) rather than the slow ramping rate of 0.2°C min^-1^ (Table 4). The use of a slow ramping rate (i.e., 0.2°C min^-1^) to measure species’ CT_max_ resulted in a failure to forecast their activities in response to ground temperatures (see: foraging temperature section 3.4 and Fig 4). In addition, when species were exposed to temperatures corresponding to their CT_max_ measured at this slow ramping rate in the static assays, most species remained active even after long periods of exposure (> 10 min., static assay section 3.3 and Fig 3), suggesting that those temperatures were not representative of species’ critical limits [see 36 for Critical Thermal Maximum]. A biologically relevant CT_max_ of a species should force individuals of that species to seek thermal refuges as soon as possible, and therefore correspond to a relatively short exposure duration during a static assay.

For instance, when exposed to very hot temperatures in the field (67–70°C), individuals of the Saharan Silver Ant (*Cataglyphis* genus) limit their foraging activities to approximately 10 minutes before returning to thermal refuges [37, 38]. A period of several minutes should represent a critical duration for individual ants to be exposed to their thermal limits, as heterogeneous habitats provide ample opportunities for individuals to locate thermal refuges. Our static assays also revealed high heterogeneity in the CT_max_ of individual ant species, with some individuals of some species remaining active even after being exposed to temperatures corresponding to their CT_max_ for over 30 minutes. Except in a few completely open habitats (e.g., deserts), such long durations of exposure are unlikely to be a major constraint on the foraging activities of small ectotherms. Furthermore, in the static assays, the ant species showed important variation in their exposure duration-based tolerance under temperatures corresponding to their CT_max(0.2)_ (Fig 1B and Fig 3). If species’ CT_max(0.2)_ were used to compare their thermal limits, the 24 species exposed to their CT_max(0.2)_ temperatures in the static assays would display high interspecific variation in exposure duration-based tolerance (S4 Table in S1 Appendix). Such interspecific variation in exposure duration-based tolerance was substantial, even between species that displayed the most similar CT_max_ values across dynamic essays using different ramping rates. For instance, while *A*. *risii* and *Ooceroea biroi* displayed similar CT_max (0.2)_ values in dynamic assays (at 39.8°C and 40.1°C, respectively), they differed extensively in their exposure duration-based tolerances of these temperatures in static assays; *O*. *biroi* could tolerate 40.1°C for 17.7 minutes but *A*. *risii* could only tolerate 39.8°C for 2.3 minutes (S4 Table in S1 Appendix). In contrast, when species were exposed to temperatures corresponding to their CT_max (0.5)_ and CT_max (1)_ in the static assays, less variation in their exposure duration-based tolerance was observed, allowing for a more direct comparison of their upper thermal limits (Fig 3).

Some studies measured environmental temperature of some species and have justified the use of slow ramping rates by referring to temperature fluctuation of some species micro-habitat [5, 7, 14, 15], and proposed that in interspecific comparisons of ectotherms, measurements of species’ upper thermal limits should relate to their thermal niches [46]. However, when slower (as compared to faster) ramping rates are used in dynamic assays, species are usually exposed to increasing temperatures for longer periods of time before reaching their upper thermal limits [5]. We therefore recommend the use of ramping rates on the basis of exposure duration-based tolerance to examine ramping rate effects and the measured CT_max_ (Fig 1A & 1B) including static assays (Fig 3) and a comparison with field data on observed foraging temperatures when available (Table 2, Fig 4).

Ideally, a species’ CT_max_ should correspond to the temperature that induces a heat-coma in individuals of that species [25, 36]. This conceptualization of CT_max_ will provide a functional trait that is useful for interpreting species’ use of habitats and microhabitats. Our results show that the ramping rates used in dynamic assays of ant species’ CT_max_ can directly shape the relationships observed among the CT_max_ of different species assemblages that are classified based on habitats or microhabitats in linear mixed models (Table 4). In particular, the ramping rates used in measurements of CT_max_ can strongly influence conclusions about the differences between the upper thermal limits of species from urban habitats and those from forest habitats. Such methodological issues can bias general conclusions about the ecology of species based on species’ CT_max_ in future studies.

### 4.3 Foraging temperature maximum

Foraging temperature maximum (FT_max_) is one of the most intuitive measures of species’ upper thermal limits. It is derived from field observations and the identification of a maximum temperature threshold after which individuals of a species suspend their foraging activity [37]. Here, we examined species’ behaviors in the field and compared those with their CT_max_ values retrieved using different ramping rates. Although the concepts of CT_max_ and FT_max_ both relate to species’ upper thermal limits, they are markedly different. CT_max_ is supposed to represent a species’ maximum physiological threshold [36]; and thus it is expected that a species’ CT_max_ should exceed its FT_max_, at which individuals cease to actively forage. While a species’ FT_max_ is affected by both its abiotic and biotic interactions [39], its CT_max_ is not. At a temperature exceeding a species’ CT_max_, individuals of that species should lose muscle control, and display an onset of spasms and heat-shock [5]. If a species’ CT_max_ is substantially lower than its FT_max_, it can likely forage at temperatures exceeding its CT_max_; this represents a biological underestimation of a species’ thermal limit under laboratory conditions. Our results show discrepancies between species’ FT_max_ and CT_max_ values, which are most extensive when the slowest ramping rate (0.2°C min^-1^) is used to measure CT_max_. At this ramping rate, six species (out of 7) display CT_max_ values that are 4.4°C to 8.1°C lower than their FT_max_. Such gaps question the biological relevance of using a slow ramping rate to measure CT_max_. In contrast, the majority of CT_max_ values retrieved using the fastest ramping rate (1°C min^-1^) aligned more precisely with species’ FT_max_ values (Fig 4 and Table 2).

The ramping rate used affects the values of CT_max_ measured and the forecasting of species’ activities. Therefore the use of a reliable CT_max_ is paramount and should refer to a biologically relevant thermal limit. Using an unreliable CT_max_ may result in a mischaracterization of the activity patterns and distribution of a species. We illustrate this problem with the case of the Red Imported Fire Ant, *S*. *invicta*, which has established populations in Hong Kong following introductions from the USA [47]. The CT_max_ of this species in the USA has been measured with the use of slow ramping rates such as 0.12 or 0.2°C min^-1^ [16, 48] as well as a faster ramping rate of 1.0°C min^-1^ [16, 49]. Coincidently, our observations of the foraging activity of this species in the field (N = 1,398) suggest a thermal threshold (FT_max_) that corresponds with its CT_max_ measured at 1.0°C min^-1^ in both the USA and in Hong Kong (this study), with a marginal difference of 0.71°C on average. In comparison, the species’ FT_max_ exceeds values of its CT_max_ measured at slower ramping rates by an average of 4.77°C. While few intensive studies of ectotherm species’ activity-temperature relationships—such as those of *S*. *invicta*—are available [48, 50, 51], our field observations of this species demonstrate the importance of identifying biologically relevant CT_max_ values that can predict species’ activity patterns. Such approaches are not only important for understanding the ecology of individual species but also for characterizing whole assemblages (Table 2, Fig 3).

Measuring FT_max_ is challenging because it is difficult to control the environmental conditions and to control for the effects of biotic interactions such as competition, which may ultimately affect the values measured [52]. Including comparisons between CT_max_ and FT_max_ in our CT_max_ framework (Fig 1) allow to capture field and biological relevance of upper thermal limit. Often, the habitat in which a species is most encountered is unlikely to experience temperatures that are close to that species’ thermal limit; this is especially true for species living in the leaf-litter layer of closed-canopy forests. For instance, the lowest CT_max_ value we observe (independently of the ramping rate used) among the ant species from Hong Kong is 38.3°C (i.e., a soil/litter-dwelling ant, *Strumigenys minutula*). This temperature remains 0.5°C higher than the highest air temperature recorded in Hong Kong’s history (Hong Kong observatory). Moreover, in the absence of direct solar radiation, soil temperatures are similar or lower than air temperatures [53]. Thus, for some habitats or microhabitats, air temperatures measured in the field cannot be used directly as maximum temperature thresholds of the species present and the microclimates they experience. Knowledge of microclimates is paramount for understanding species’ thermal niches [54]. Our observations of ant species’ foraging activity patterns across a wide range of surface temperatures (i.e., 10–66.2°C) provide crucial information on the microclimates that these species experience. Therefore, measurements of species’ upper thermal limits using experimental approaches (i.e., dynamic assays for CT_max_) remain necessary so long as they can estimate biologically relevant thresholds for these limits.

### 4.4 Phylogenetic signals

The methods used to measure eco-physiological traits can strongly influence the values retrieved, bias empirical findings, and cause ecological phenomena to be misinterpreted. Our study shows that CT_max_ is strongly affected by the ramping rate used, and that the use of a specific ramping rate use can induce significant biases in subsequent analyses of species’ phylogenetic relationships (Table 3). A number of studies have shown that upper thermal limit is phylogenetically conserved in ectotherms, such as in ants [34, 55], fruit flies [56], and lizards [57]. In contrast, other studies retrieved no evidence for a relationship between phylogeny and thermal tolerance. Notably, a slow ramping rate of 0.2°Cmin^-1^ was used in these studies [28, 58]. To the best of our knowledge, our study represents the first to compare phylogenetic signal in species’ CT_max_ to the ramping rate used in CT_max_ measurements. Our results show that the detection of a phylogenetic signal (i.e., Pagel’s *λ* and Bloomberg’s *K*) is directly influenced by the methodology used to measure CT_max_ (Table 3). The findings also show that apart from differences in the topography of the phylogenetic tree and the species pool, differences in ramping rate could also explain these inconsistencies of the phylogenetic signals. Although upper thermal limit has been shown to be strongly constrained by evolutionary history [55], the ramping rates used to collect the data should also be considered as an important cofounding factor in tests for various evolutionary hypotheses. In such analyses, one should avoid the use of a slow ramping rate (e.g., 0.2°C min^-1^) to measure CT_max_ values (at least for ants), as well as the combination of CT_max_ values originating from different methodologies.

### 4.5 Predicted CT_max_ from biophysical model vs. experimental CT_max_ from physiological measurement

Recent studies have provided a mathematical model, based on thermal tolerance landscapes, to predict the CT_max_ value for a given species independently of the ramping rate used [11, 12]. As mentioned by the authors [12], this model has been developed based on the study of eleven *Drosophila* species, and should therefore be tested with other ectotherms. While not central to our study, our dataset provides a good opportunity to test this model and understand its generality. Our results, however, provide rather limited support to the model, with predicted and observed values diverging substantially in most cases tested (see details results in S3 Appendix). It thus appears that the biophysical model should be used cautiously, and may not be suitable for a majority of the ant species tested here. Although this model can provide important insights into species’ ecophysiology, it has limited value in helping to identify a satisfactory ramping rate that will facilitate biologically relevant CT_max_ measurements. Ultimately, our study supports the need for further validation and examination in other ectotherm groups [12], with further research needed for identifying pertinent ramping rates in ecological studies.

## 5. Conclusion

The use of CT_max_ to study ectotherms has significantly increased in the past decades, and its application has yielded multiple predictions about the impacts of global change [17, 59, 60]. As shown here as well as in other studies of ectotherms (Ants: 13; 16. *Drosophila* fly: 12, 16), the CT_max_ values observed are, however, largely affected by the ramping rates used and identity of species used in dynamic assays. Ideally, CT_max_ values should facilitate comparisons between studies and be grounded in biological relevance. Our study thus establishes a new and hybrid method to address this goal, integrating dynamic and static assays in addition to comparisons with field data (FT_max_), to identify a reliable ramping rate for ant species. Each approach we use serves to test whether a given ramping rate produces biologically relevant CT_max_ values. Our results indicate that a ramping rate of 1.0°C min^-1^ is the most appropriate for measuring the CT_max_ of ant species. This new methodological framework can be used to detect the limitations of particular ramping rates and help to identify more reliable CT_max_ values for trait-based studies in functional ecology. In particular, studies using CT_max_ should carefully consider the ramping rate used, as our results show that different ramping rates can lead to different conclusions about relationships within and between ecological communities (Table 4) and also bias the detection of phylogenetic signal (Table 3 and Fig 5). Our results support the use of a ramping rate of 1°Cmin^-1^ over relatively slower ramping rates. This ramping rate provides a more reliable measure of ant species CT_max_ that aligns with assumptions about ant species’ thermal adaptions and observations of their foraging activities within natural environments [61]. Although we only applied the integrated framework for the three most frequently used ramping rates among studies of ant species’ CT_max_, our study represents the most comprehensive investigation of ant species’ CT_max_ thus far, and demonstrates the limitations associated with the use of particular ramping rates and their consequences for conclusions relating to species’ habitat use or phylogenetic signal. We believe that this integrated framework should also be applied for other terrestrial ectotherms. The framework we use to identify a suitable ramping rate should produce reliable CT_max_ values that hold the potential to reveal crucial information about species’ upper thermal limits. Such information will be useful for various studies on climate warming, land-use change, pest control and trait-based ecology.

## Supporting information

S1 Appendix (DOCX)

S2 Appendix (XLSX)

S3 Appendix (DOCX)
