# Supplementary material for: Testing the reliability and ecological implications of ramping rates in the measurement of Critical Thermal maximum
Source: PLoS One. 2022 Mar 14;17(3):e0265361. doi: 10.1371/journal.pone.0265361 (PMC8920270; doi:10.1371/journal.pone.0265361)
Supplement: S1 Appendix — (DOCX) [file pone.0265361.s001.docx]

**S1 APPENDIX**

**S1 Table. Ant species and their natural history.**

List of the 27 ant species used in this study and including their nesting and main foraging strata, exotic status and habitats. Strata: S = Subterranean, G = Ground-dwelling, A = Arboreal.

**S2 Table. Ant collection and climatic information.**

Ant species studied in the current study, with coordinates of their sampling sites and associated maximum air temperature (Tmax) for each locality. For Hong Kong climate (which covers a small area of 1,106 km²), we used a 30 m resolution Hong Kong climate model (Morgan and Guénard, 2018) and extracted the maximum air temperature for each species in function of the geographic coordinates of sampling location. For Macau climate (area of 32 km²), we retrieved the maximum air temperature for each species field site from the closest weather stations of Macau SAR (https://www.smg.gov.mo/en/, data retrieved in 1^st^ Oct 2021). Lat. = latitude; Long. = longitude; Tmax = Air temperature maximum.

Morgan B, Guénard B. New 30 m resolution Hong Kong climate, vegetation, and topography rasters indicate greater spatial variation than global grids within an urban mosaic. Earth System Science Data. 2019;11(3):1083-98. <https://doi.org/10.5194/essd-11-1083-2019>.

**S3 Table.** **Summary table for CT_max_ and ramping rate in dynamic assays.**

Summary table of dynamic assay, mean and SD of CT_max_ values in different ramping rates measured, ANOVA, and Tukey’s test. Temp. = Thermal treatment, Diff = differences between the maximum and minimum response values (the CT_max_ value). Thermal treatment is specific for ramping rate group and increase the temperature based on the ramping rate group (0.2, 0.5, and 1.0 °C min^-1^).

**S4 Table. Summary table for temperature treatment and thermal tolerance duration in static assays.**

Summary table for static assay mean and SD of duration in different temperature treatments measured, Kruskal-Wallis test = KW test and Dunn’s test. Temp. = Thermal treatment, Diff = differences between the maximum and minimum response values (the exposure duration-based tolerance at the temperature set as their CT_max_ values). Thermal treatment is specific for each species and ramping rate group (0.2, 0.5, and 1.0 °C min^-1^), and the treatment is the average of their CT_max_ values.

**S5 Table. Linear regression model for each species in dynamic assays**

Linear regression model for each species in dynamic assay (CT_max_ predicted by ramping rate), all the *p*-values are significant differences (< 0.05) with the exception of *Anochetur risii* resulting in higher CT_max_ values with fast ramping rate used.

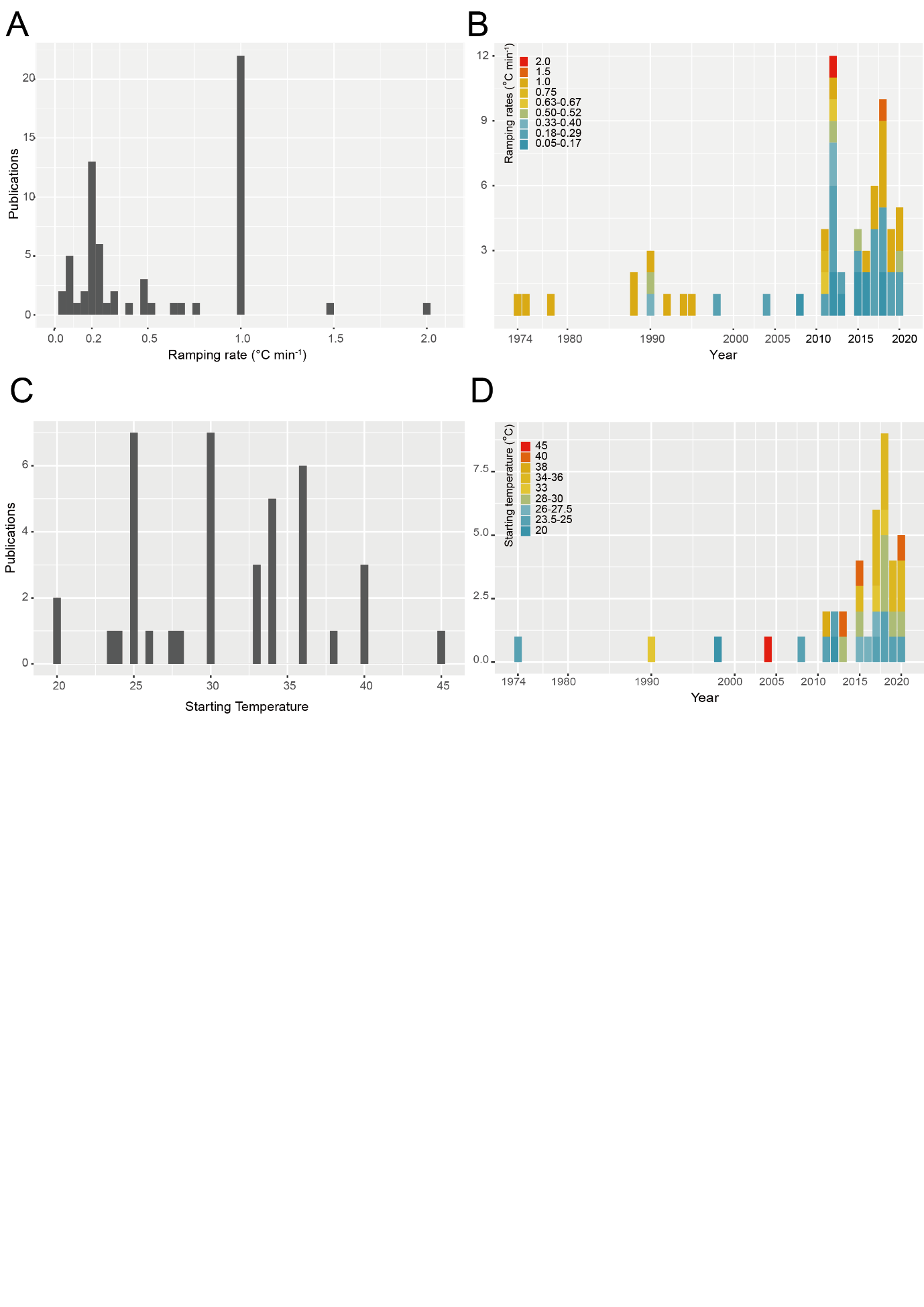


**S1 Fig: Review of dynamic CT_max_ studies from 1974 to 2020 June in ants (Formicidae).**

(A) The number of publications in function of the ramping rates used and (B) years of publications and (C) in function of the starting temperatures used in experiments (C) and (D) years of publication.


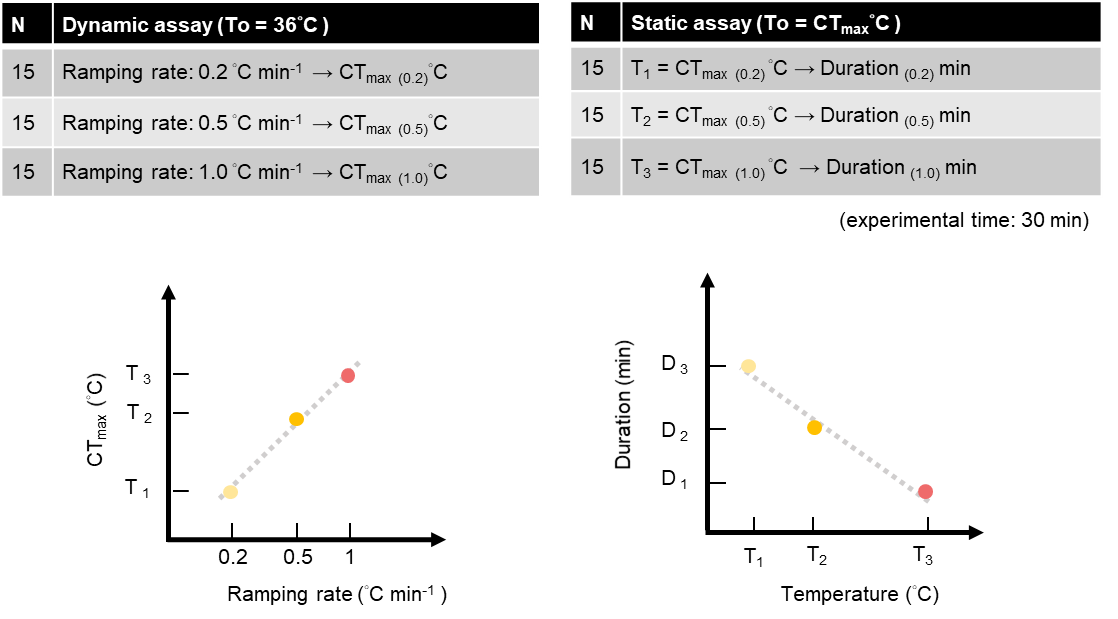


**S2 Fig: Experimental setting of the dynamic and static assays.**

Left: The experimental diagram of dynamic assay in this study with three treatments of ramping rate. Right: The experimental diagram of static assay in this study with three temperature treatments (CT_max(0.2)_, CT_max(0.5)_ and CT_max (1.0)_.


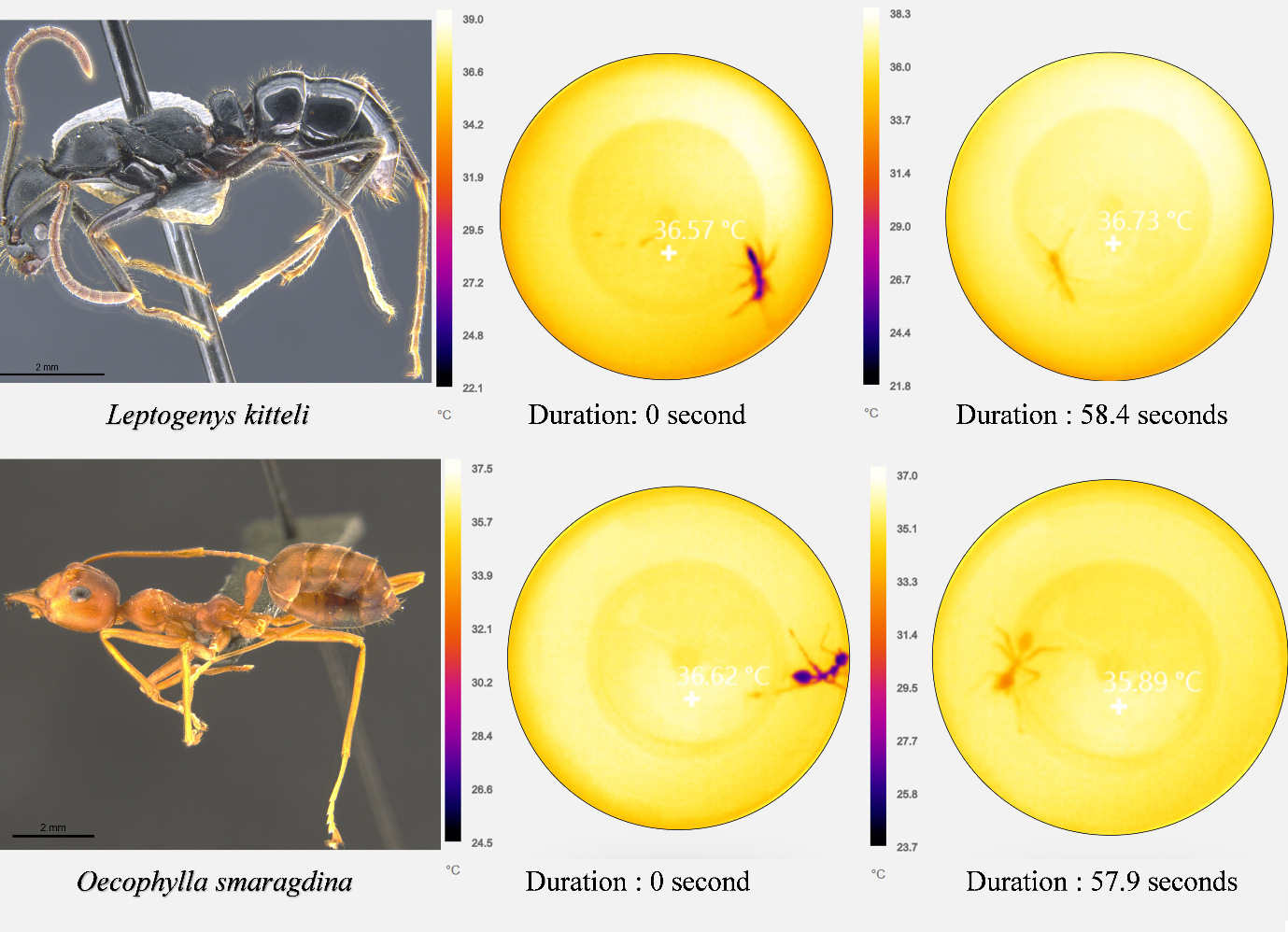


**S3 Fig: Body surface temperature changes in the thermal assays.**

Preliminary tests of the *Leptogenys kitteli* and *Oecophylla smaragdina* body surface temperature changes when they put in the 36 °C environments from the 24-25 °C room temperature. The results shown the ant body temperatures raised 9-10°C within one minute resulting in the ramping rate of ants as 9-10 °C min^-1^. The tests used the digital dry bath (Benchmark - BSH1004) and an additional infrared camera (Fluke Ti480P).

_
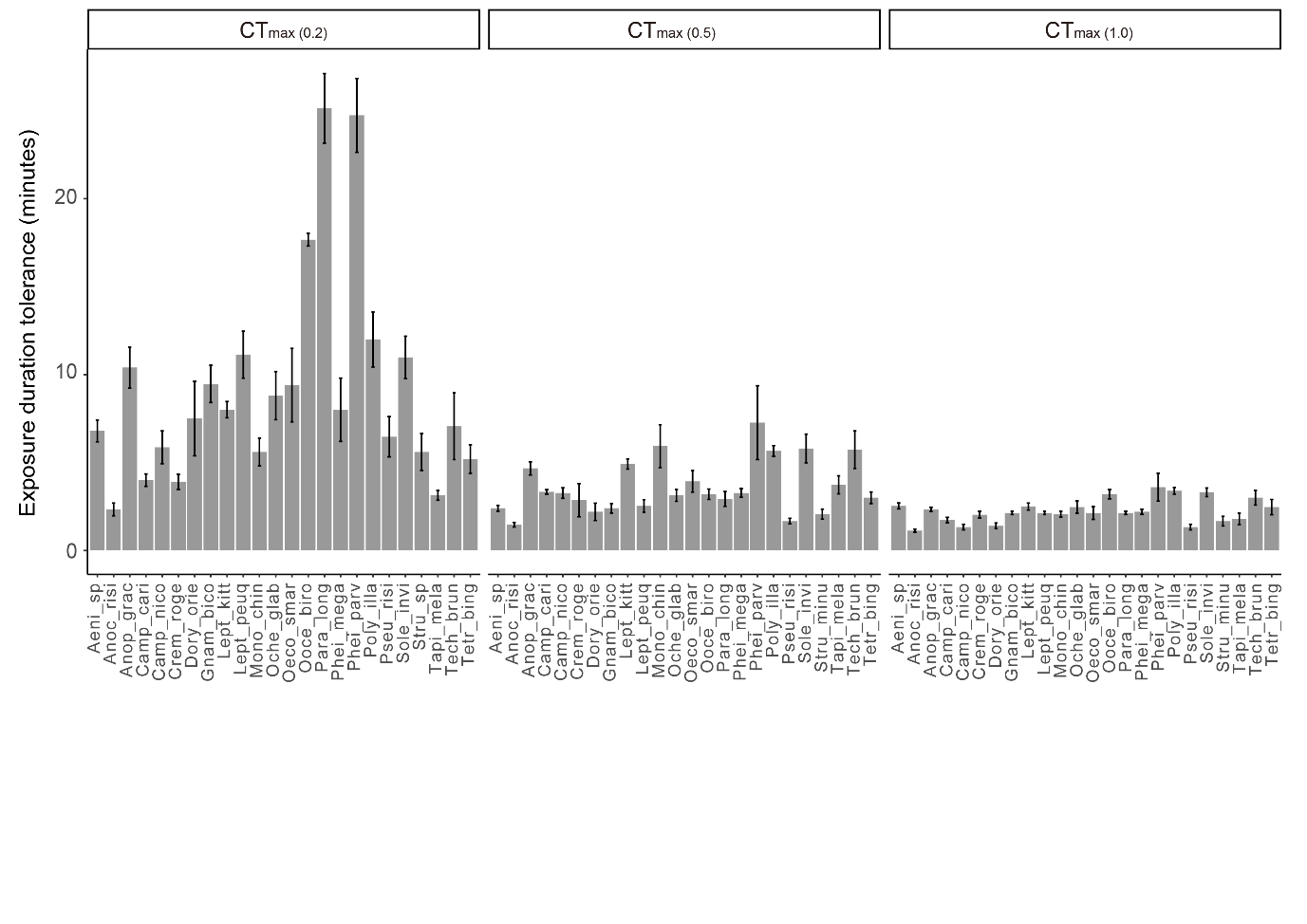
_

**S4 Fig.** Exposure duration-based tolerance of each ant species in the static assays.

Mean exposure duration-based tolerance values and their variation (error bars refer to ±SE) of 24 ant species during static essays in function of the threshold temperatures retrieved from the three treatments, CT_max (0.2, 0.5, and 1. 0)_ obtained during dynamic treatments. The 24 species in static assays show high heterogeneity in the species-specific CT_max (0.2)_ treatment and more homogeneity in those of CT_max (0.5 and 1.0)_. Overall, all species present the highest similarity of exposure duration-based tolerance for the CT_max (1.0)_ treatment. Abbreviation of species name is presented in Table S1.


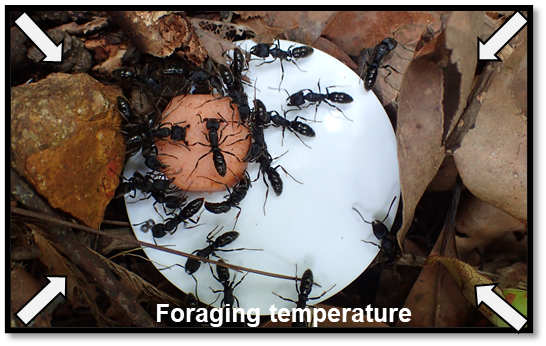


**S5 Fig: Measuring foraging temperature for ants.**

Methodology used to measure foraging temperature around baiting station, each record of foraging temperature is calculated as the average of four measurements taken around each baiting station (indicated by arrows) and associated to the species active on bait at that time.


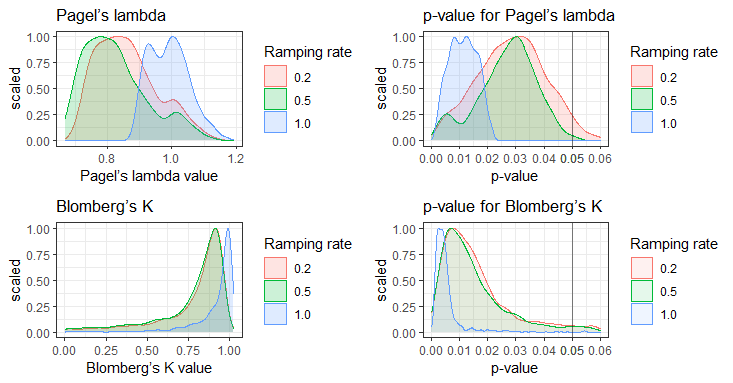


**S6 Fig:** **Phylogenetic signals of CT_max_ obtained from 0.2, 0.5, and 1 °Cmin^-1^ ramping rates.**

Pagel’s lambda and Blomberg’s K for the 27 ant species in the present study. High values of Pagel’s lambda and Blomberg’s K indicate high phylogenetic signals for CT_max_ values and p-value < 0.05 is the significance level.
