# Supplementary material for: Testing the reliability and ecological implications of ramping rates in the measurement of Critical Thermal maximum
Source: PLoS One. 2022 Mar 14;17(3):e0265361. doi: 10.1371/journal.pone.0265361 (PMC8920270; doi:10.1371/journal.pone.0265361)
Supplement: S3 Appendix — (DOCX) [file pone.0265361.s003.docx]

**S3 APPENDIX**

**Recent Critical Thermal Maximum (CT_max_) biophysical model not suitable to ants**

Recent studies have shown that the CT_max_ values of a given species could be predicted for any ramping rate treatments based on the exposure tolerance duration and static temperature treatment [1, 2]. These studies were based on biophysical theory and heat damage to develop predicted CT_max_ model, and a well-known and high prediction model developed by Rezende et al. (2020) using 11 *Drosophila* species as study organisms for validation [2]. As the authors [2] wrote *“Although here we assume that body temperature equals ambient temperature, which is reasonable for Drosophila (*[*21*](about:blank#pill-R21)*), for other ectotherms whose thermal inertia is not negligible body temperatures can be estimated from biophysical modeling and ambient temperature records. However, empirical validation in other taxa remains necessary.”*. So, further CT_max_ studies using other organisms are needed for research focusing on ramping rates effects and CT_max_ measurements.

In order to examine the biophysical model and its use in ants, this study also tested the biophysical model with our data. Rezende et al. (2020) used the data from Jorgensen et al. (2019) with the previous study [3] to develop a biophysical model. We have used the data generated from our study (24 species from 2,791 individuals) to apply the model and R scripts based on two R functions (i.e. *tolerance.landscape* and *dynamic.landscape*; Rezende et al. 2020) to predict the CT_max_ values. The results obtained, however, showed low predictive power, in contrast to the original data presented with the model, with a predictive power (R^2^) of 96% [2]. For ants, however, half of the species CT_max_ values could not be predicted by the thermal landscape and the survival model from Rezende et al. (2020). Our data shown that ants may follow the limitations discussed by Rezende et al. (2020), that not all ectotherms’ CT_max_ values are suitable in their biophysical formula.

Overall, the predictions of the model we obtained show "strong" predictions of the CT_max_ values for 17% to 50% of the species, with an average of 35% (see Table S1 with detailed results below). In addition, mediocre results, or deviating from 1° to 3°C from observed values, were obtained in another 35% of the modelling results (ranging from 21 to 46% depending of the ramping rate considered); while poor results, deviating by more than 3°C and as much as 11.4°C, represented 31% of the results obtained (25 to 37.5%). Although three linear regressions are significantly positive, the R^2^ values obtained have rather limited explanatory power (0.3, 0.41 and 0.48 for 0.2, 0.5 and 1°C min^-1^ ramping rates respectively, Fig. S1), and are clearly different from the results obtained with the *Drosophila* model, and which were restricted to ramping rates varying from 0.05 to 0.2° C per minute (with R^2^ = 0.962; Rezende et al. 2020). Thus, a majority of the predicted CT_max_ values obtained appear rather inaccurate and not fully predicting experimental biological data.

In conclusion, the biophysical model does not appear to be suitable for a majority of ant species tested here, and the generality of the models need to be further evaluated using data from other taxa.

**Table S1.** **Predicted CT_max_ vs. experimental CT_max_ in ants**

Differences between predicted CT_max_ and experimental CT_max_ in function of three ramping rates (0.2, 0.5, and 1°C min^-1^). The differences highlighted in yellow refer to rather poor results obtained with the biophysical model.

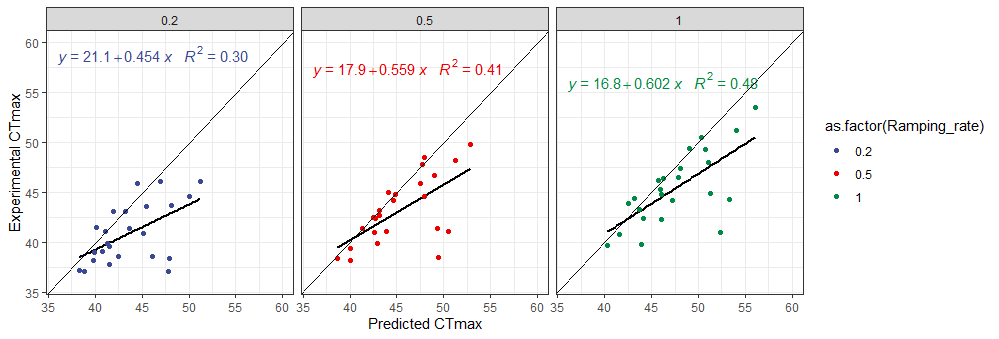


**Fig S1. Predicted CT_max_ vs. experimental CT_max_ in ants**

Predicted CT_max_ (°C) vs. experimental CT_max_ (°C) retrieved in three different ramping rates (0.2, 0.5, and 1°C min^-1^). Reference lines (thin black line) showing where predicted CT_max_ = experimental CT_max_. *R^2^* = determination coefficient.

**References:**

1. Jørgensen LB, Malte H, Overgaard J. How to assess *Drosophila* heat tolerance: Unifying static and dynamic tolerance assays to predict heat distribution limits. Funct Ecol. 2019;33(4):629-42. [https://doi.org/doi:10.1111/1365-2435.13279](about:blank).

2. Rezende EL, Bozinovic F, Szilágyi A, Santos M. Predicting temperature mortality and selection in natural Drosophila populations. Science. 2020;369(6508):1242-5. [https://doi.org/10.1126/science.aba9287](about:blank).

3. Rezende EL, Castañeda LE, Santos M. Tolerance landscapes in thermal ecology. Funct Ecol. 2014;28(4):799-809. [https://doi.org/10.1111/1365-2435.12268](about:blank).
